# Caffeine increases maximal fat oxidation during a graded exercise test: is there a diurnal variation?

**DOI:** 10.1186/s12970-020-00400-6

**Published:** 2021-01-07

**Authors:** Mauricio Ramírez-Maldonado, Lucas Jurado-Fasoli, Juan del Coso, Jonatan R. Ruiz, Francisco J. Amaro-Gahete

**Affiliations:** 1 Department of Physiology. Faculty of Medicine, University of Granada, Av. Conocimiento S/n, 18011 Granada, Spain; 2 PROFITH “PROmoting FITness and Health Through Physical Activity” Research Group, Department of Physical Education and Sport, Faculty of Sport Sciences, University of Granada, Granada, Spain; 3 Centre for Sport Studies, Rey Juan Carlos University, Madrid, Spain

**Keywords:** Exercise performance, Body composition, Nutrition, Body weight, Dietary supplement

## Abstract

**Background:**

There is evidence that caffeine increases the maximal fat oxidation rate (MFO) and aerobic capacity, which are known to be lower in the morning than in the afternoon. This paper examines the effect of caffeine intake on the diurnal variation of MFO during a graded exercise test in active men.

**Methods:**

Using a triple-blind, placebo-controlled, crossover experimental design, 15 active caffeine-naïve men (age: 32 ± 7 years) completed a graded exercise test four times at seven-day intervals. The subjects ingested 3 mg/kg of caffeine or a placebo at 8 am in the morning and 5 pm in the afternoon (each subject completed tests under all four conditions in a random order). A graded cycling test was performed. MFO and maximum oxygen uptake (VO_2max_) were measured by indirect calorimetry, and the intensity of exercise that elicited MFO (Fat_max_) calculated.

**Results:**

MFO, Fat_max_ and VO_2max_ were significantly higher in the afternoon than in the morning (all *P* < 0.05). Compared to the placebo, caffeine increased mean MFO by 10.7% (0.28 ± 0.10 vs. 0.31 ± 0.09 g/min respectively, *P* < 0.001) in the morning, and by a mean 29.0% (0.31 ± 0.09 vs. 0.40 ± 0.10 g/min, *P* < 0.001) in the afternoon. Caffeine also increased mean Fat_max_ by 11.1% (36.9 ± 14.4 [placebo] vs. 41.0 ± 13.1%, *P* = 0.005) in the morning, and by 13.1% (42.0 ± 11.6 vs. 47.5 ± 10.8%, *P* = 0.008) in the afternoon.

**Conclusion:**

These findings confirm the previously reported diurnal variation in the whole-body fat oxidation rate during graded exercise in active caffeine-naïve men, and indicate that the acute ingestion of 3 mg/kg of caffeine increases MFO, Fat_max_ and VO_2max_ independent of the time of day.

**Trial registration:**

NCT04320446. Registered 25 March 2020 - Retrospectively registered

## Introduction

Endurance performance has been traditionally understood as a multifactorial concept in which maximal oxygen uptake (VO_2max_), ventilatory thresholds and muscular efficiency play important roles [1]. Considerably less attention has been paid, however, to the importance of the management of substrate oxidation during prolonged exercise and its relationship with endurance performance [1]. Metabolic flexibility, known as the capacity to adapt fuel utilization to substrate availability, has recently been suggested an additional key factor affecting performance in endurance disciplines [2]. Given that maximal fat oxidation during a graded exercise test (MFO), and the intensity of exercise that elicits MFO (Fat_max_), have been recognized as potential determinants of metabolic flexibility during exercise [3, 4], it seems plausible that both MFO and Fat_max_ strongly influence endurance performance. Certainly, higher fat oxidation rates (at the expense of lower carbohydrate use) at moderate exercise intensities might help spare endurance athletes’ muscle and liver glycogen stores during training and competition [5].

In athletes, it is known that endurance performance is poorer early in the morning and late at night compared with the afternoon [6], and that MFO and Fat_max_ are higher in the afternoon compared to the morning whether in non-athlete male students [7], in untrained normal-weight and obese individuals [8], or in endurance-trained athletes [9]. The difference has been explained by the higher body temperature, the enhanced neural activation and contractile properties of the skeletal muscle, and the higher plasma catecholamine concentrations found in response to exercise in the afternoon compared to the morning and evening [10, 11].

Caffeine is a natural alkaloid used by both endurance and resistance athletes as an ergogenic aid [12, 13]. It does not appear in the World Anti-Doping Agency’s 2004 list of prohibited substances. Interestingly, the urine caffeine concentration recorded in doping control tests, especially for athletes of endurance-based sports, has increased progressively since it was removed from the above list [14]. Certainly, low-to-moderate doses of caffeine (~ 3–9 mg/kg) [15] can increase endurance performance [16] via the induction of significant increases in VO_2max_, peak pulmonary ventilation, and muscle oxygen saturation during submaximal workloads [17, 18]. A recent study by Gutierrez-Hellín et al. [19] also shows caffeine ingestion to increase the MFO in healthy subjects of both sexes. Similarly, the ingestion of 5–7 mg/kg of caffeine during steady-state aerobic exercise seems to increase the utilization of fat as a fuel in detriment to the use of carbohydrate [20–22]. Preliminary reports also suggest that caffeine intake may help counteract the diurnal variation observed in exercise performance [6, 23–26]. Mora-Rodríguez et al. [24] reported that the acute ingestion of caffeine reverses the time-of-day reduction seen in maximum dynamic strength and muscle power output in resistance-trained men, while Boyett et al. [23] report that trained athletes are more likely to obtain ergogenic effects from caffeine in the morning than the evening (at least in terms of cycling performance). It would be of interest to know whether caffeine attenuates the diurnal variation seen in both the rate of whole-body fat oxidation during exercise, and in endurance performance, and whether caffeine has a synergistic effect with the already known diurnal variation in energy metabolism. The aim of the present work was, therefore, to investigate the effect of caffeine intake on the diurnal variation of MFO and Fat_max_ during a graded exercise test in active men. Based on the available scientific literature, we hypothesised that: (i) The acute ingestion of caffeine will increase MFO, Fat_max_ and VO_2max_ independent of the time of day. (ii) There will be a diurnal variation in MFO, Fat_max_ and VO_2max_, with values being higher in the afternoon than in the morning.

## Methods

### Subjects

Fifteen active men, aged 32 ± 7 years, volunteered to participate in the current study (clinicaltrials.gov; NCT04320446). To be included all subjects had to: (i) have a body mass index of 18.5–28 kg/m^2^, (ii) be non-smokers, (iii) suffer no disease that might be aggravated by physical exercise, (iv) take no medication or drugs, (v) be naive caffeine consumers (< 50 mg/day), (vi) have previous experience in endurance training (i.e., self-reporting of at least 2 years of endurance training including three or more training sessions/week [3.6 ± 0.2 sessions/week]), (vii) be free of any caffeine allergy, and (viii) have incurred no musculoskeletal injury during the previous month. All subjects were recruited by social networks and local media, and they provided oral and written informed consent before their enrolment. Procedures were performed in accordance with the latest revised Declaration of Helsinki (2013). The University of Granada Research Ethics Committee approved the present project (N° 507/CEIH/2018).

### Design and methodology

**Fig. 1:**
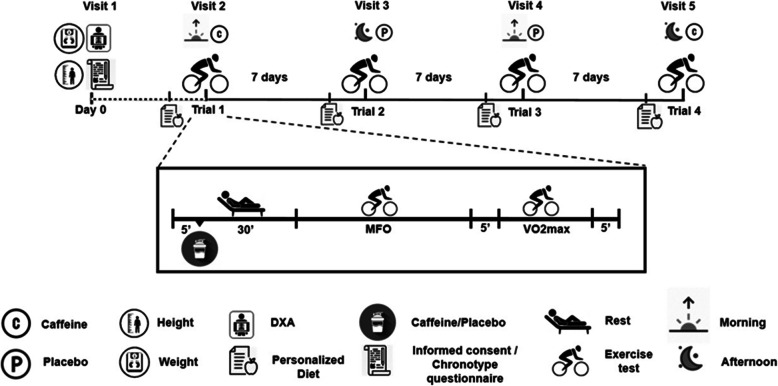
Study procedures. Abbreviations: DXA; dual energy X-ray absorptiometry test

This study had a triple-blind (i.e. participants, evaluation staff and statistician), placebo-controlled, crossover experimental design involving a graded exercise test performed by all subjects on four occasions, with each occasion separated by 7 days (Fig. 1). They were asked to maintain their physical activity levels and nutritional habits during the intervention. Subjects ingested either a dose of 3 mg/kg anhydrous caffeine in powder form (the extract of HSN® green coffee beans [Harrison Sport Nutrition (HSN) Store, Granada, Spain]) or a 100% pure microcrystalline cellulose placebo [Acofarma, Madrid, Spain]) 30 min before each test. Both supplements were unflavoured, uncoloured and odourless. The use of the above-mentioned dose was based on the results of previous studies reporting caffeine to be effective at increasing fat oxidation during exercise in trained athletes [19]. Both the caffeine and placebo were dissolved in 250 ml of water and served in opaque, indistinguishable recipients; the subjects were therefore blind to what they had received.

The study was performed between June and November 2019. Measurements were conducted between 8 and 11 am (providing MFO-morning, Fat_max_-morning, and VO_2max_-morning), and between 5 and 8 pm in the afternoon (providing MFO-afternoon, Fat_max_-afternoon, and VO_2max_-afternoon). The order of (i) the time of the day when the exercise tests were performed, and (ii) the administration of caffeine or placebo, were randomized using a function included in MS Excel for Windows®. However, all subjects were tested under all ingestion/time-of-day condition combinations.

Before testing began (Day 0), subjects’ weight and height were recorded using a Seca model 799 electronic column scale and stadiometer (Seca, Hamburg, Germany), and their body mass index calculated as weight divided by the square of the height (kg/m^2^). The body weight measured on this day was used in the dosage calculations for the entire experiment. Subjects were asked to be barefoot and to wear only light clothing during these measurements. Dual energy X-ray absorptiometry, performed using a Hologic Discovery Wii device (Hologic, Bedford, MA, USA), was conducted to determine subject lean and fat mass (kg). All subjects also completed the HÖME questionnaire to determine their chronotype (i.e., morningness–eveningness). They were subsequently categorized as (i) definite evening type (score range 16–30), moderate evening type (31–41), neither type (score 42–58), moderate morning type (59–69) and definite morning type (70–86) [27]. Finally, all subjects were provided instructions: (i) to avoid moderate and vigorous physical activity 24 and 48 h respectively before test days, (ii) to adhere to a standardized, personalized diet (50% carbohydrates, 30% fat and 20% protein) during the 24 h before each test day and to keep to the same meal order independent of the time of the day at which the test was performed, (iii) to arrive at the laboratory in a motorized vehicle to avoid physical activity, and (iv) to fast for 3 h before arrival. Compliance with these instructions was checked by self-reported dietary and exercise records.

On test days, a personalized dose of caffeine (3 mg/kg) or placebo was provided before performing the graded exercise test - undertaken using a Cardgirus Medical Pro cycle ergometer (C&G Innovations, Cochin, India) under controlled environmental conditions (temperature: ranged from 22 to 24 °C and humidity: ranged from 40 to 50%). After substance intake, subjects rested in the supine position for 30 min to ensure absorption. Thereafter, a submaximal graded exercise test was begun. This consisted of cycling at 50 W maintaining a cadence of 60–100 rpm for 3 min (warm-up protocol), with subsequent 25 W increments of the workload every 3 min until reaching a respiratory exchange ratio of 1.0 [3, 28]. They then rested for 5 min with free access to water before beginning a maximal graded exercise test to measure their VO_2max_. This began with the same warm-up protocol, followed by increments of 50 W every minute until self-reported exhaustion [29]. Indirect calorimetry data were registered using a CPX Ultima CardiO2 breath-by-breath gas analyzer (Medical Graphics Corp., St. Paul, MN, USA). A prevent™ metabolic flow sensor (Medgraphics) fitted to a model 7400 oronasal mask (Hans Rudolph Inc., Kansas City, MO, USA) was used to obtain respiratory data. Simultaneously, a Polar RS800 heart-rate monitor (Polar Electro Inc., Woodbury, NY, USA) was used to monitor the heart rate during both maximal and submaximal graded exercise. The gas analyzer was calibrated immediately before each graded exercise, according to the manufacturer’s recommendations.

#### Submaximal graded exercise test

The VO_2_ and VCO_2_ data derived from the last 60 s of each graded exercise stage were taken into account [30]. Fat oxidation rates were estimated from the stoichiometric equation of Frayn, assuming urinary nitrogen excretion to be negligible [31]. MFO and Fat_max_ were determined by plotting fat oxidation values (dependent variable) against the relative exercise intensity (independent variable) to construct a third degree polynomial regression curve for each subject (0,0) from a graphical depiction of fat oxidation values as a function of exercise intensity [32].

#### Maximal graded exercise test

The criteria for deeming VO_2max_ to have been reached were: (i) attaining a steady (increase < 2 ml/kg/min) in VO_2_ despite a further increase in workload, (ii) showing a maximal heart rate between 10 bpm above and below the age-predicted maximum [33], and (iii) reaching a respiratory exchange ratio of > 1.1 [34]. When these criteria were not met, peak oxygen consumption was taken into account (i.e., the highest VO_2_ value measured over the last 60 s of the test).

### Statistical analysis

Sample size and power calculations were determined based on the results of a prior study [9]. We considered MFO differences between (i) morning vs. afternoon and (ii) caffeine vs. placebo test in order to assess the sample size requirements for the two-way analysis of variance (time-of-the day x substance). As a result, we expected to detect an effect size of 0.05 g/min considering a type I error of 0.05 with a statistical power of 0.90 with a minimum of 12 participants. Assuming a maximum loss of 20%, we decided to recruited a total of 15 participants. The results of every test were blindly introduced into the SPSS v.22.0 package (IBM Corporation, Pittsburgh, PA, USA); analyses were also performed blind to experimental conditions. Visual check histograms, Q-Q plots and Shapiro-Wilk tests were used to check the normality of all variables. Since all study outcomes were normally distributed, parametric tests were used to examine differences between conditions. Two-way analysis of variance (*time-of-the day x substance*) was used to compare MFO, Fat_max_ and VO_2max_ under different study conditions. When a significant F value was obtained, a Bonferroni post hoc analysis was performed to determine pairwise differences. Additional analyses were conducted after adjusting for age, chronotype, lean mass and fat mass. Finally, experimental conditions with a common characteristic (i.e., morning vs. afternoon, and caffeine vs. placebo) were grouped to independently calculate the effect of the time of the day and substance provided on MFO, Fat_max_ and VO_2max_ using pairwise tests. Significance was set at *P* < 0.05. Lastly, we also calculated the standardized effect sizes using Cohen’s d coefficients. Graphs were plotted using GraphPad Prism 5 (GraphPad Software, San Diego, CA, USA).

## Results

Table 1 shows the characteristics of the study participants. The chronotype was homogeneously distributed (*n* = 5 moderate evening type, *n* = 5 neither type, and *n* = 5 moderate morning type).

**Table 1:** Characteristics of the study subjects (*n* = 15)

Age (years)	32.4	±	7.2
Weight (kg)	79.9	±	10.7
Height (m)	1.8	±	0.1
Body mass index (kg/m^2^)	25.6	±	2.3
Fat mass (%)	18.5	±	3.9
Lean mass (kg)	61.7	±	9.0
HÖME questionnaire score			
Definitive evening type (n [%])	0 [0.0]		
Moderate evening type (n [%])	5 [33.3]		
Neither type (n [%])	5 [33.3]		
Moderate morning type (n [%])	5 [33.3]		
Definite morning type (n [%])	0 [0.0]		

Values expressed as means ± standard deviation

**Fig. 2:**
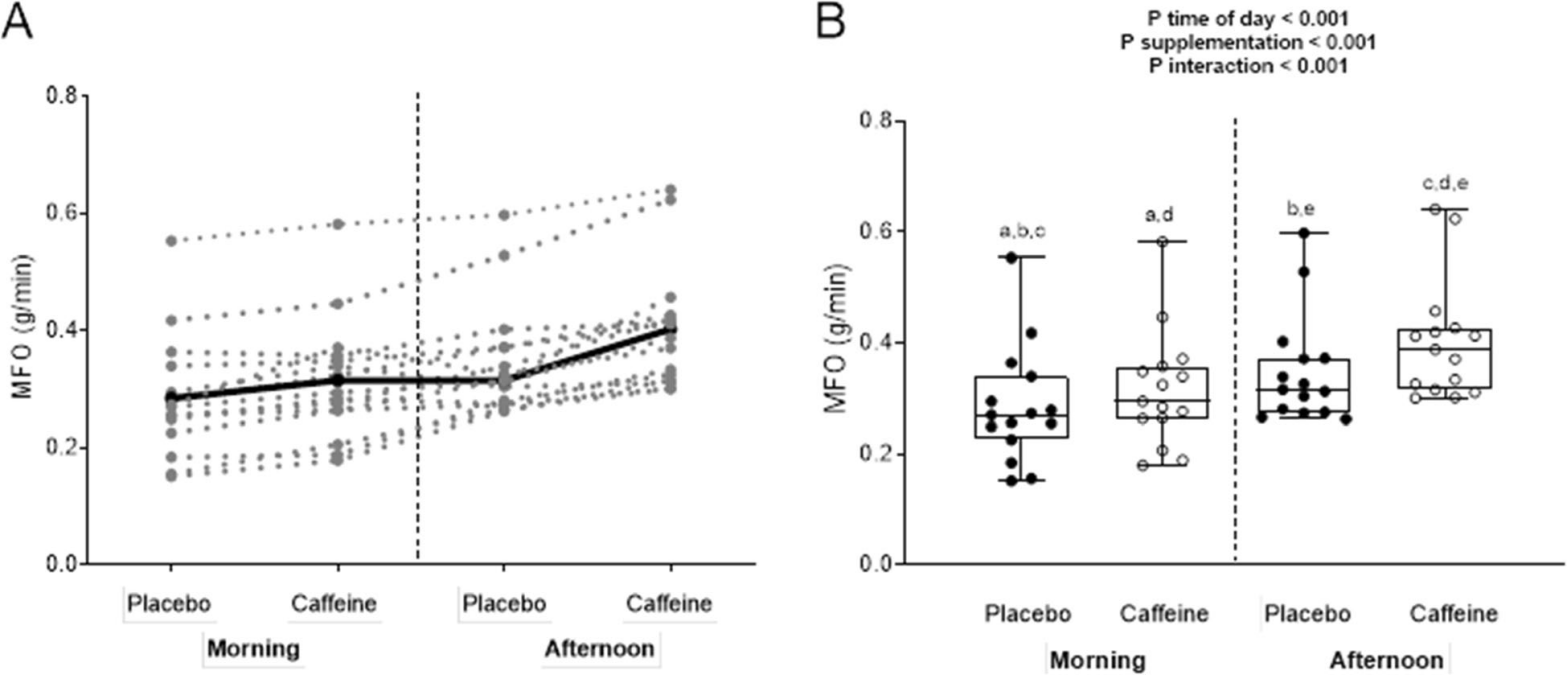
Maximal fat oxidation (MFO) in the morning, and in the afternoon, after ingestion of caffeine or the placebo. Panel **a**: Individual observations for each subject (grey lines), and the mean for all subjects (black line). Panel **b**: Individual observations for each subject (black dots), standard deviation and minimum/maximum values (box-and-whisker plots), and the *P* value obtained by two-way ANOVA. Similar letters (i.e. a-a; b-b, etc.) indicate significant post hoc differences

Time-of-day had a significant effect on MFO (*P* < 0.01), with the latter always higher (ranging from 10.7 to 29.0%) in the afternoon than in the morning. Compared to the placebo, caffeine intake increased mean MFO by 10.7% in the morning (0.28 ± 0.10 vs. 0.31 ± 0.09 g/min respectively, *P* < 0.001; d = 0.32; Fig. 2) and by 29.0% in the afternoon (0.31 ± 0.09 and 0.40 ± 0.10 g/min, *P* < 0.001; d = 0.95; Fig. 2). A significant *time-of-the day x substance* interaction was observed in MFO (*P* < 0.001; Fig. 2).

**Fig. 3:**
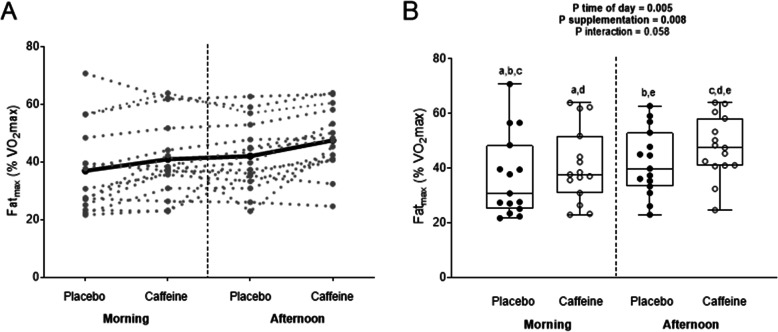
Intensity of exercise eliciting maximal fat oxidation (Fat_max_) in the morning, and in the afternoon, following the ingestion of caffeine or the placebo. Panel **a**: Individual observations for each subject (grey lines), and the mean for all subjects (black line). Panel **b**: Individual observations for each subject (black dots), standard deviation and minimum/maximum values (box-and-whisker plot), and the *P* value obtained by two-way ANOVA. Similar letters (i.e., a-a; b-b, etc) indicate significant post hoc differences

Time-of-day also had a significant effect on Fat_max_ (all *P* < 0.01), which was always higher (ranging from 11.1 to 13.1%) in the afternoon than in the morning. Compared to the placebo, caffeine intake increased Fat_max_ by 11.1% in the morning (36.9 ± 14.4 vs. 41.0 ± 13.1% respectively; d = 0.30; Fig. 3) and by 13.1% in the afternoon (42.0 ± 11.6 vs. 47.5 ± 10.8%, respectively; d = 0.49; Fig. 3). A strong trend toward significance *time-of-the day x substance* interaction was observed in Fat_max_ (*P* = 0.058; Fig. 3).

**Fig. 4:**
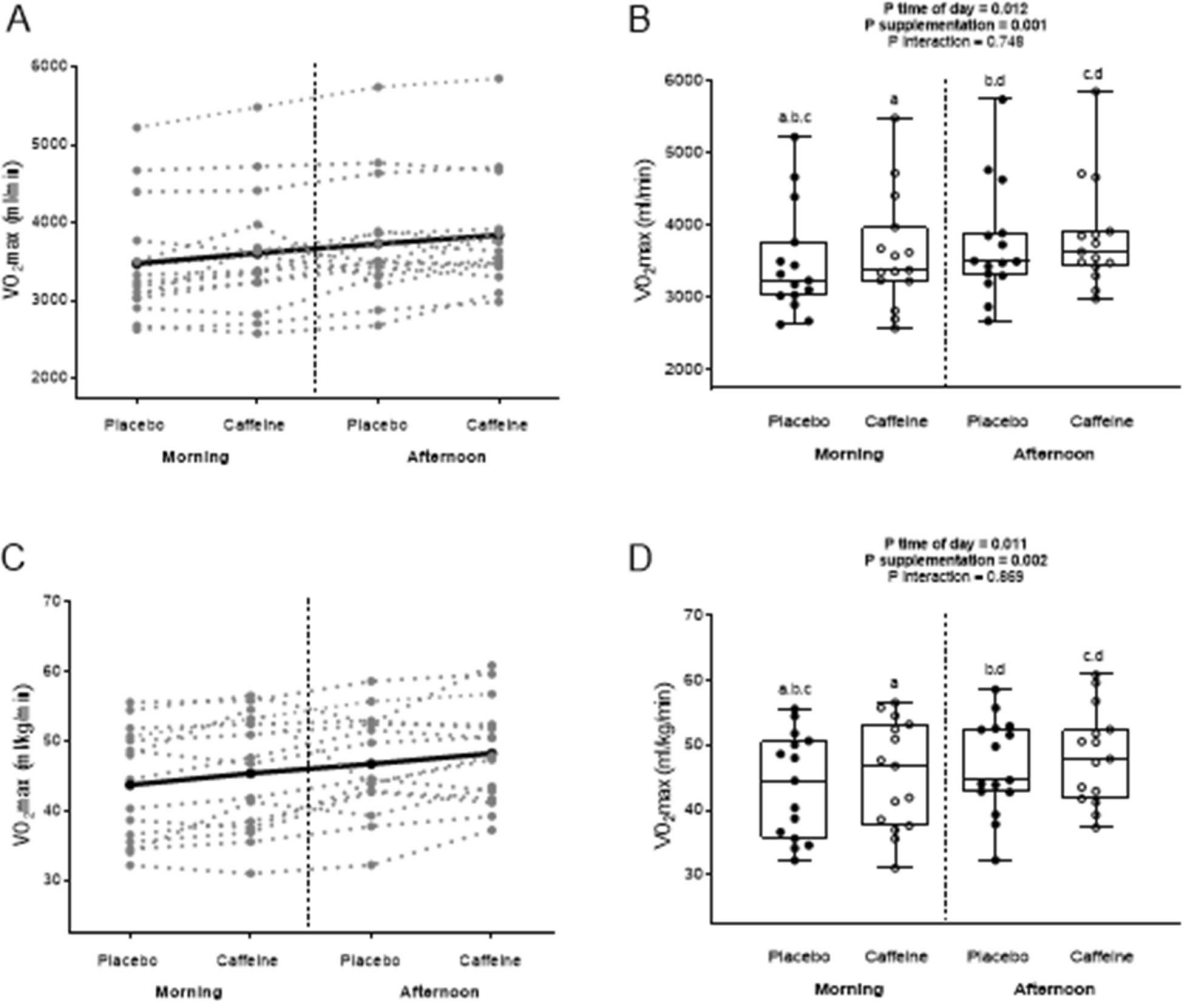
Maximum oxygen uptake (VO_2max_) in absolute terms in the morning, and in the afternoon, following the ingestion of caffeine or the placebo. Panel **a**: Individual observations for each subject (grey lines), and the mean for all subjects (black line). Panel **b**: Individual observations for each subject (black dots), standard deviation and minimum/maximum values (box-and-whisker plot), and *P* value obtained by two-way ANOVA. Similar letters (i.e. a-a; b-b, etc) indicate significant post hoc differences. VO_2max_ relative to weight in the morning, and in the afternoon, following the ingestion of caffeine or the placebo. Panel **c**: Individual observations for each subject (grey lines), and the mean for all subjects (black line). Panel **d**: Individual observations for each subject (black dots), standard deviation and minimum/maximum values (box-and-whisker plot), and the *P* value obtained by two-way ANOVA. Similar letters (i.e. a-a; b-b, etc.) indicate significant post hoc differences

Finally, time-of-day had a significant effect on VO_2max_ (*P* < 0.05), which was always higher (ranging from 3.2 to 3.9%) in the afternoon than in the morning. Compared to the placebo, caffeine intake increased VO_2max_ by 3.9% in the morning (43.7 ± 7.8 vs. 46.7 ± 7.0 ml/kg/min, respectively; d = 0.40; Fig. 4) and by 3.2% in the afternoon (45.4 ± 8.0 vs. 48.2 ± 7.0 ml/kg/min; d = 0.37; Fig. 4). No significant *time-of-the day x substance* interaction was observed in VO_2max_ (*P* > 0.7; Fig. 4).

All the significant differences reported above persisted after adjusting for age, chronotype, lean mass and fat mass (data not shown).

## Discussion

The present results indicate that caffeine intake increases MFO and Fat_max_ as well as VO_2max_ independent of the time of day. The highest values for these variables were all obtained in the afternoon after caffeine intake. The results also show that, in the morning, the values of MFO after caffeine ingestion are nearly equivalent to those recorded in afternoon tests without caffeine supplementation. This suggests that caffeine increases whole-body fat oxidation during graded exercise in the morning to a value similar to that seen without caffeine in the afternoon. Overall, these results suggest that a combination of acute caffeine intake and exercise at moderate intensity in the afternoon provides the best scenario for individuals seeking to increase whole-body fat oxidation during aerobic exercise.

### Diurnal variation of MFO, Fat_max_ and VO_2max_

The present findings provide further evidence regarding the diurnal variation of MFO and Fat_max_, which have been reported higher in the afternoon than in the morning [7–9]. It should be noted that these previous studies were conducted using a treadmill graded exercise test to measure these variables. In the present work, a cycloergometer graded exercise test was used. Thus, together, these results suggest that the diurnal variations in MFO and Fat_max_ are independent of subject characteristics and of the ergometer and protocol used to assess the whole-body fat oxidation rate during exercise. A number of studies have reported athletes to show better endurance performance during the afternoon than the early morning and late evening [10, 35], a finding with which the present results agree. However, in one study conducted in trained male athletes, no differences in VO_2max_ were seen between the morning and the afternoon [9]. With respect to this particular variable, the discrepancy might be explained by the different ergometers used (i.e., a cycloergometer in the present work, and a treadmill in the latter work), or the different biological characteristics of the study subjects, or the different fasting times before conducting the exercise test (3 h vs. 7–10 h respectively).

Endurance performance peaks in the afternoon usually coinciding with the highest core body temperature reached during the day [36]. This temperature increases energy metabolism, improves muscle compliance, and facilitates actin-myosin cross bridging [11]. Moreover, the exercise-induced catecholamine peak is higher in the afternoon than in the morning [10, 11]. This catecholamine release promotes an increase in lipolysis in both skeletal muscle and adipose tissue [11, 35], raising the plasma fatty acid content and explaining the higher fat oxidation rates observed in the afternoon. Since the present work collected no data on body core temperature or catecholamine release during exercise, further studies will be needed if these variables are to be better linked to the physiological mechanisms behind the observed diurnal variation in VO_2max_, MFO and Fat_max_.

### Caffeine ingestion, MFO, Fat_max_ and VO_2max_

The results of the current study support the use of caffeine as an ergogenic aid to raise fat oxidation during exercise, as well as to increase VO_2max_, and agree with the findings of previous investigations showing that caffeine improves fuel oxidation during prolonged exercise [20–22] and enhances endurance performance [12]. The present results also agree with those obtained by Gutiérrez-Hellín et al. [19] who reported 3 mg/kg caffeine to increase MFO in healthy active young men, as well as those reported by Dodd et al. [37] who indicate that 5 mg/kg of caffeine improved VO_2max_ in naive caffeine consumers. The higher MFO, Fat_max_ and VO_2max_ values recorded in the present work after caffeine ingestion may be explained by (i) an enhancement of fatty acid mobilization and oxidation, aided by an increase in the release of epinephrine, (ii) a blockage of the A_1_, A_2A_, and A_2B_ adenosine receptors, thus promoting the release of acetylcholine and dopamine which dampens pain perception, blunts perceived exertion, and delays fatigue [16, 38–40], (iii) an increase in motor unit recruitment, which results in higher rates of muscular contraction and alertness [16], and/or (iv) an increase in muscle oxygen saturation that might facilitate the use of fat at moderate exercise intensities and lead to higher VO_2_ values at maximal exercise intensity [18].

### Effects of caffeine intake on the diurnal variation in MFO, Fat_max_ and VO_2_max

Mora-Rodríguez et al. [24] reported the acute ingestion of caffeine (3 mg/kg) to reverse the morning reduction in muscle performance - in fact to allow comparable muscle performance to those seen in the afternoon. These findings suggest that caffeine ingestion in the morning could be used by athletes as an ergogenic aid to help them avoid morning-induced reduction in muscle performance. In addition, Boyett et al. [23], who investigated whether the effect of caffeine on athletes’ performance in a 3 km cycling time trial was influenced by the time of day and training status, concluded that caffeine enhanced cycling performance more in the morning than in the evening. These findings are partially in line with those of the present study, suggesting that acute caffeine intake before exercise serves as an effective ergogenic aid for reversing morning-induced reductions in resistance exercise performance and endurance-like performance.

The present study suffers from the limitation that body temperature and blood variable data were not collected during the graded protocol test, precluding any confirmation that metabolic and hormonal variables play a role in the diurnal variation of MFO, Fat_max_ and/or VO_2max_. Moreover, we did not control the sleep quality and quantity of the participants. Further, the present study was performed in active men; the results cannot, therefore, be directly extrapolated to women or sedentary populations, etc. Finally, the sample size was relatively small.

### Practical applications

- Caffeine intake increases MFO and Fat_max_ as well as VO_2_max independent of the time of day.
- The highest values for these variables were all obtained in the afternoon after caffeine intake.
- Caffeine increases MFO in the morning to a value similar to that seen without caffeine in the afternoon.
- A combination of acute caffeine intake and exercise at moderate intensity in the afternoon provides the best scenario for individuals seeking to increase MFO.

## Conclusions

In summary, the acute ingestion of caffeine (3 mg/kg) 30 min prior to a graded exercise test increased the MFO, Fat_max_ and VO_2max_ in active caffeine-naïve men independent of the time of day. Further, the existence of a diurnal variation in MFO, Fat_max_ and VO_2max_ was confirmed, with values for all being higher in the afternoon than in the morning. The present findings also support the notion that caffeine ingestion in the morning helps to increase MFO and Fat_max_ levels during exercise in the afternoon. These results support the use of caffeine as an ergogenic aid during training or competition during the morning. The combination of acute caffeine intake and exercise at moderate intensity in the afternoon seems to be the best scenario for individuals seeking to increase the amount of fat utilized during continuous aerobic exercise. Whether higher doses of caffeine induce greater effects on whole-body fat oxidation during graded exercise tests and further improves endurance performance remains to be investigated.

## Data Availability

The datasets used and/or analysed during the current study are available from the corresponding author on reasonable request.

## Abbreviations

VO_2max_: Maximal oxygen uptake
MFO: Maximal fat oxidation during a graded exercise test
Fat_max_: Intensity of exercise that elicits maximal fat oxidation during exercise
